# The association of psychological stress and health related quality of life among patients with stroke and hypertension in Gaza Strip

**DOI:** 10.1186/1744-859X-5-6

**Published:** 2006-05-19

**Authors:** BT Baune, Y Aljeesh

**Affiliations:** 1 Department of Psychiatry, School of Medicine, James Cook University, Australia; 2 Department of Psychiatry and Psychotherapy, University of Munster, Germany; 3 Islamic University, Gaza, Palestinian Authority

## Abstract

**Background:**

The study was performed to investigate the association of psychological stress and quality of life (QOL) among patients with the cardiovascular disease (CVD) of hypertension plus stroke or hypertension only.

**Methods:**

The WHOQOL-BREF questionnaire was applied to 112 hypertensive patients with hypertension plus stroke and 224 hypertensive patients without stroke. Psychological stress was assessed with SCL-90. Means scale scores were compared using student-t-test and predictors of QOL were calculated with covariance analysis.

**Results:**

Patients with stroke had a significant lower QOL than patients without stroke and a significantly higher level of stress (p < 0.01). In analyses of covariance psychological stress was significantly correlated to all domains of QOL among non-stroke patients. The same psychological and sociodemographic factors showed little impact on the stroke patients in these multivariable analyses. In these models psychological stress had a significant impact on the global domain of QOL among stroke patients. Income and gender were the only sociodemographic factors being significantly associated with the physical (education) and social (gender) domains of QOL in stroke patients.

**Conclusion:**

Psychological stress was strongly correlated with all domains of QOL in patients without stroke and was only partly associated with QOL among patients with stroke. Future studies should investigate if psychological stress is a factor suitable for educational and psychological interventions aiming at stress reduction in CVD patients which might substantially contribute to better health related quality of life in these patients.

## Background

Quality of life (QOL) is a phrase often used in health care settings at policy and administration levels, in clinical assessments of therapies, and in clinical management of individual cases. While QOL is a broad concept that covers such areas as social, environmental, economic, and health satisfaction, health-related quality of life (HRQL) is less wide ranging, including mental and physical health and their consequences. Health-related quality of life (HRQL) is considered as one of the key concepts in contemporary practice of medicine and delivery of health care [1-4]. Quality of life assessment is complicated by the fact that there is no universally accepted definition for QOL. The researchers of this study have adopted the WHO definition of QOL which identifies QOL as a multidimensional concept. It is defined as "individuals' perceptions of their position in life in the context of the culture and value system in which they live and in relation to their goals, standards, and concerns" [5].

In general, HRQL can be influenced directly and indirectly by various disease related factors. Chronic diseases, such as cardiovascular diseases and mental disorders (e.g. depression) potentially decrease short-term and long-term HRQL. Direct effects of the disease itself and side effects of the treatment may influence HRQL in patients with stroke or hypertension. Hypertension and stroke are two prevalent medical conditions which may affect quality of life substantially. Moreover, anti-hypertensive medication may induce fatigue, dizziness, and sexual dysfunction, which in turn impairs patient's activity of daily living and quality of life [6-8]. Sexual dysfunction is not necessarily caused by the hypertensive drug therapy, but is likely due to other factors such as vascular disease [9].

Major medical consequences of stroke concern different physical and psychological aspects such as loss of sense, palsy, disturbance of body image, depression and change in patient's role also affecting HRQL. Despite of side effects of medication (e.g. antidepressant, anti-cholinergic, and anti-adrenergic agents) potentially leading to decreased well-being [10], a meta-analysis of well-selected and comparable trials has shown the positive impact of anti-hypertensive medication on patient's quality of life as a whole despite disturbing side effects [11]. Grimm et al. showed in a study with patients suffering from mild hypertension that anti-hypertensive medication improves quality of life secondary to a reduction of blood pressure [12].

The assessment of HRQL in this study is based on a holistic and multidimensional approach of HRQL, which includes several dimensions such as physical, socioeconomic, spiritual and psychological aspects. Stress is among the psychological factors potentially influencing QOL [3,4]. Moreover, mental well-being is essential part of the QOL concept and therefore part of the assessment of quality of life carried out with e.g. the SF36 instrument or the WHOQOL questionnaire [5]. In relation to cardiovascular diseases, psychological factors such as stress seem to play an even more important role for patients' HRQOL suffering from stroke or hypertension than patient characteristics, blood pressure, or drug-related factors [14].

Psychological stress has been targeted in intervention and rehabilitation programs among patients with hypertension, because it is believed that stress reduction can improve hypertension [15]. Certain intervention programs for stress reduction aimed successfully at a reduction of blood pressure [15-17].

Despite the positive impact of medication on quality of life in hypertensive patients suffering from stroke or hypertension only recent research suggests paying more attention to psychological factors such as stress accounting for quality of life among various cardiovascular disorders, because psychological stress seems to be an additional factor suitable for interventions aiming at improved HRQL for these patients [15].

However, little is known about the association of psychological stress and health related quality of life among patients with various cardiovascular conditions. To further investigate the relationship of psychological stress and quality of life among these patients, we carried out a study among patients either with hypertension only or hypertension plus stroke in Gaza.

### Objectives

This study investigates the impact of psychological stress on health related quality of life (HRQL) among patients with or without stroke in Gaza Strip.

### Study design

A one to two matched case-control study was carried out. The case group consists of 112 subjects with stroke and history of hypertension, and the control group consists of 224 subjects with hypertension only. Cases and controls were matched by age, sex, starting point of therapeutic regimen for both pharmacological and non-pharmacological treatment, time of hypertension, enrollment location of hospital related health care clinics and calendar time. For each case two identical controls were recruited to detect small differences between the two groups [18] and to compensate potential loss of controls.

### Patients and methods

Cases and controls were recruited from the same geographical area in Gaza Strip [8,10]. Three main hospitals in Gaza Strip (Shefa, Nasser, and Khan Younis hospital) and the geographically and administratively related primary health care clinics were pinpointed for the selection of the study population. Each selected primary health care clinic was associated with one of the three hospitals in relation to diagnostic and treatment as well as follow-up procedures. The population under study was aged between 35 and 69 years. The lower age limit was used, because hypertension and stroke considerably occur among people above 34 years of age in Gaza. The upper limit was chosen because this is the average life expectancy in Gaza Strip. Inclusion and exclusion criteria of the study will be explicitly given below in the manuscript. The study was approved by the Ministry of Health in Gaza and the local ethical committee in Gaza Strip.

### Inclusion and exclusion criteria

All available discharge data of patients from the selected hospitals were screened for cases. Patients who had been hospitalized for acute stroke and history of hypertension between 1^st ^January and 31^st ^December 2001 were defined as cases (N = 180).

In total 112 (62%) hypertensive patients with stroke were ultimately selected from the registers. The diagnosis of stroke was confirmed by a physician and a head CT scan was performed. Stroke was defined as "a sudden loss of brain function resulting from disruption of the blood supply to a part of the brain" [2]. A computed tomography head scan had been carried out in patients with hypertension plus stroke. Both groups were selected only, if a history of other physical diseases (diabetes, myocardial infarction, atrial fibrillation, pulmonary oedema, asthma) was excluded. Sixty-eight (38%) out of 180 selected cases were excluded from the study: 19 (28%) died after first stroke; eighteen (26%) had a second stroke; seven subjects (10%) were over 69 years old; 10 (15%) participated in the pilot study; seven (10%) patients had no computer tomography head scan; four (6%) refused to participate in the study; three (4%) patients started their therapeutic regimen less than one year.

Controls were defined as having hypertension only, without history of stroke prior to interview. Hypertension was defined when the threshold levels of 140 mm Hg systolic and 90 mm Hg diastolic were reached at three different independent measurements within one week, repeated twice over a 2 months period prior to the diagnosis of hypertension. Further grading into mild, moderate, severe hypertension was not made.

Controls may have developed stroke later on after the study period. The occurrence of potential later development of stroke in controls after the interview was studied for the period of the study by telephone follow-up interview and clinic visits. During the period of the study no one of the controls developed a stroke.

These patients with a diagnosis of hypertension received health care follow-up because of anti-hypertensive medication in eight governmental primary health care clinics. Controls were selected from the same primary health care clinics where cases used to receive follow-up appointments for hypertension on primary health care level before hospital admission due to the development of stroke.

### Questionnaires

The first part of the questionnaire gives information on age, gender, marital status, education and socio-economic status. Quality of life for the period 2 weeks prior to the interview was assessed with the brief version of the WHO Quality of life questionnaire [12,13]. Psychological stress two weeks prior to the interview was assessed using five psychological items with a 5-level scale ranging from none to very strong (0–4) adapted from the Symptom Check List by Derogatis (SCL-90) with regards to (1) nervousness, (2) paranoia, (3) social phobia, (4) criticism and (5) loneliness [19]. According to the evaluation of the SCL-90 items, high scores on each of these psychological factors express psychological stress. As no cut-off-points exist for psychological stress measured with the SCL-90, low stress is indicated by numbers pointing to '0' and high psychological stress is characterized by numbers pointing to '20'. The sum score (scale from 0–20) was used as a continuous variable in the analysis of covariance.

The short version of the WHO Quality of life (WHOQOL-BREF) assessment gives means of four single domains (physical, psychological, social, and environmental) and one overall domain (global value). The range of each domain of QOL is from 0–100. As no cut-points exist for good and poor QOL measured by WHOQOL-BREF, poor quality of life is indicated by numbers pointing to '0' and high quality is characterized by numbers pointing to '100'.

A certified translation of the questionnaire from English into Arabic language including a backward translation was performed twice independently, and the results were checked for inconsistencies.

### Statistical analysis

Frequency tables were calculated to describe the study population regarding sex, age, level of stress and quality of life. The statistical significance of differences between groups was tested with Mann-Whitney-U-test (tables 2 and 3). Each quality of life domain was correlated with the continuous variable psychological stress by the use of Spearman's correlation coefficient (table 3).

**Table 1:** Gender and age among stroke and non-stroke patients

	**Stroke (N = 112)**			**Non-stroke (N = 224)**		
	N			N		

**Gender**						
Female	54			108		
Male	58			116		
**Age**		Male %	Female %		Male %	Female %
40–45	3	33.3	66.7	6	33.3	66.7
46–51	18	55.6	44.4	36	55.6	44.4
52–57	32	53.1	46.9	64	53.1	46.9
58–63	32	56.3	43.7	64	56.3	43.7
64–69	27	44.4	55.6	54	44.4	55.6

**Table 2:** Education and average income in $ per month among stroke and non-stroke patients

	**Stroke (N = 112)**	**Non-stroke (N = 224)**	Mann-Whitney-U-test
	n, (%)	n, (%)	*p*-value

**Level of education**			
No education	67 (59.8)	84 (37.5)	
Primary school	11 (9.8)	29 (12.9)	
Preparatory school	8 (7.1)	44 (19.6)	
Secondary school	15 (13.4)	41 (18.3)	0.001
University degree	10 (8.9)	25 (11.2)	
Other	1 (0.9)	1 (0.4)	
Total	112 (100)	224 (100)	

**Average income per month in U.S. $**			
Less than 200 U.S. $	48 (42.9)	101 (45.1)	
200–350 U.S. $	29 (25.9)	76 (33.9)	
360–550 U.S. $	29 (25.9)	39 (17.4)	
560–750 U.S. $	6 (5.4)	8 (3.6)	0.236
More than 750 U.S. $	-	-	

Total	112 (100)	224 (100)	

**Table 3:** Clinical and sociodemographic factors and psychological stress by domains of quality of life

	**Domains of quality of life **^d^				
**Characteristics**	**Physical **Mean (SD)	**Psychological **Mean (SD)	**Social **Mean (SD)	**Environmental **Mean (SD)	**Global **Mean (SD)

Diagnosis					
Stroke	41.26 (15.49)***	40.47 (15.31)***	51.04 (17.37)***	46.04 (17.41)***	45.2 (23.14)***
Non-stroke	67.45 (15.69)	64.76 (17.19)	71.25 (15.03)	61.71 (15.26)	65.46 (18.58)
Gender					
Male	61.99 (19.38)***	61.09 (19.97)***	68.15 (17.98)***	60.35 (17.86)***	62.36 (21.51)***
Female	55.25 (19.95)	51.9 (19.28)	60.59 (18.25)	52.33 (16.41)	54.78 (22.60)
Education ^a^					
Low	54.84 (19.31)***	53.24 (18.50)***	61.87 (17.66)***	52.59 (16.18)***	55.45 (21.83)***
High	68.93 (17.83)	65.59 (21.56)	71.42 (18.85)	66.63 (17.25)	67.20 (21.49)
Income ^b^					
Low	54.46 (18.45)***	51.15 (19.01)***	61.41 (17.61)***	51.20 (16.43)***	54.78 (22.02)***
High	62.15 (20.42)	61.05 (19.98)	66.98 (18.82)	60.69 (17.43)	61.83 (22.14)
Psychological stress ^c^	-0.339 ***	-0.247 ***	-0.231 ***	-0.245 ***	-0.284 ***

p-value of Mann-Whitney-U-test: * not significant; ** p < 0.05; *** p < 0.01;

^a ^Level of education (highest degree) was classified into two groups: low level = 'no education' or 'primary school' or 'preparatory school'. high level = 'secondary school' or 'University degree';

^b ^Level of income per month: low level = income < 200 U.S. $ per month; high level = income of >200 U.S. $ per month;

^c ^Spearman's correlation coefficient of the continuous variables stress and domains of QOL; p-value of correlation coefficient: * not significant; ** p < 0.05;

^d ^range of scale of domains of QOL: 0 = poorest QOL; 100 = best QOL;

Finally, analysis of covariance was carried out for each single domain of QOL separate for patients with hypertension plus stroke and with hypertension only (tables 4 and 5). In these separate covariance models the QOL domains served as the dependent variables and age (continuous variable), gender, education (5-level categorical variable), income (5-level categorical variable) and psychological stress (continuous variable with a scale ranging from 0–20) served as covariates. For all statistical procedures SPSS statistical software, version 12.0 was used.

**Table 4:** Analysis of covariance for the impact of psychological stress, demographic and social factors on domains of quality of life among patients with hypertension

**Domains of QOL**	**Physical**		**Psychological**		**Social**		**Environmental**		**Global**	
**Factors**	F-value (df)	p	F-value (df)	p	F-value (df)	p	F-value (df)	p	F-value (df)	p

Stress	9.3 (1)	0.00	5.2 (1)	0.02	7.9 (1)	0.005	5.4 (1)	0.02	4.6 (1)	0.03
Age	0.09 (1)	0.77	0.04 (1)	0.83	0.5 (1)	0.48	0.24 (1)	0.63	0.1 (1)	0.75
Gender	2.6 (1)	0.11	16.1 (1)	0.00	9.4 (1)	0.002	5.02 (1)	0.03	4.02 (1)	0.04
Education	4.5 (5)	0.00	2.9 (5)	0.02	2.9 (5)	0.01	3.5 (5)	0.00	0.93 (5)	0.47
Income	5.7 (3)	0.00	5.1 (3)	0.00	1.7 (3)	0.16	9.1 (3)	0.00	3.5 (3)	0.02

F-value = value of covariance analysis; df = degree of freedom; p = level of significance

**Table 5:** Analysis of covariance for the impact of psychological stress, demographic and social factors on domains of quality of life among patients with stroke

**Domains of QOL**	**Physical**		**Psychological**		**Social domain**		**Environmental**		**Global**	
**Factors**	F-value (df)	p	F-value (df)	p	F-value (df)	p	F-value (df)	p	F-value (df)	p

Stress	2.9 (1)	0.09	0.00 (1)	0.99	0.08 (1)	0.88	1.8 (1)	0.21	5.3 (1)	0.02
Age	1.7 (1)	0.21	2.6 (1)	0.10	0.07 (1)	0.83	0.7 (1)	0.42	1.6 (1)	0.12
Gender	2.8 (1)	0.83	1.5 (1)	0.22	3.9 (1)	0.05	2.5 (1)	0.12	2.2 (1)	0.14
Education	1.5 (5)	0.21	1.1 (5)	0.37	0.4 (5)	0.85	2.1 (5)	0.07	0.55 (5)	0.74
Income	2.8 (3)	0.04	2.0 (3)	0.12	1.6 (3)	0.22	1.2 (3)	0.34	1.3 (3)	0.26

F-value = value of covariance analysis; df = degree of freedom; p = level of significance

## Results

### Study population

In table 1 the distribution of age and gender by diagnosis is presented. Approximately 70% of the stroke patients had a cerebral infarction and 30% a hemorrhagic infarction. The mean age of the study population was 55 years (SD 6.6). Males (51.8%) were only slightly more frequent than females in the study population (48.2%). The participants were 40–69 years old. Patients from 52 to 63 years accounted for 57% of the age distribution. One-fourth (24%) of the patients were over 63 y. Patients aged 45 years old or younger were the smallest group in the study.

### Education and income per month

The distribution of educational qualifications and of average monthly income stratified by disease status is presented in Table 2. The level of education was significantly different between patients with hypertension plus stroke and those with hypertension only (p = 0.001). Patients with hypertension were generally higher qualified than those with hypertension plus stroke. Whereas nearly 60% of patients with hypertension plus stroke presented without a formal qualification (e.g., primary, preparatory, secondary school, University degree), only a little more than one third (37.5%) among the patients with hypertension was formally not qualified. Although we found a trend that patients with hypertension only had a larger proportion of lower average monthly income compared to their counterparts, this difference was not statistically significant. Furthermore, the results indicate a financially underprivileged population under study: more than 75% of the probands had a monthly income of 350 U.S. $ or less (Table 2).

### Psychological stress, clinical, social and demographic factors

Table 3 presents mean comparisons of the domains of quality of life stratified by diagnosis, gender, education and income. Mean comparisons were made with student-t test. The results show statistically significant poorer quality of life across all domains among patients with the diagnosis of hypertension plus stroke, female gender, and among those with low education and low income compared to their counterparts. Psychological stress was statistically significant inversely correlated with the physical, psychological, social, environmental and global domains of QOL.

Age had no significant impact on any of the domains of QOL (data not shown).

### Association of psychological stress and QOL

Tables 4 and 5 present results of separate analyses of covariance estimating the impact of psychological stress and other factors on domains of QOL separate for patients with hypertension only and those with hypertension plus stroke.

Psychological stress was statistically significant correlated with all domains of QOL in patients with hypertension (Table 4). While gender was significantly correlated with the psychological, social, environmental and global domain of QOL among patients with hypertension, no such significant association was found for age. Education was significantly related to all single domains of QOL (except the global value). Income showed a significant association with the physical, psychological, environmental and global domain, but not with the social domain among patients with hypertension only (Table 4).

A different picture resulted from the analysis of covariance for patients with hypertension plus stroke (Table 5). Psychological stress was significantly correlated with the global domain only and income was significantly associated with the physical domain of QOL. Gender was significantly associated with quality of life in the social domain. The variables education and age were not significantly correlated to any of the domains of QOL among patients with hypertension plus stroke (Table 5).

## Discussion

In this study we investigated the association of psychological stress and quality of life among patients suffering from the chronic and potentially disabling cardiovascular conditions of hypertension or hypertension plus stroke.

In general, patients with hypertension plus stroke rated statistically significant lower quality of life than their purely hypertensive counterparts. In the analyses of covariance we found that psychological stress was significantly related to all domains of QOL among patients with hypertension. Contrary, psychological stress had no major effect on domains of QOL among hypertension plus stroke patients in these multivariable models.

Several other studies confirm the finding that patients with hypertension plus stroke have lower QOL than those with hypertension only due to the disabling effects of stroke [20-22]. In more general terms, Kempen et al. showed that health related quality of life is substantially affected by chronic medical morbidity, such as stroke and hypertension [22]. Especially the disabling effects of stroke may have an effect on reduced QOL even 1 year after stroke as reported by Wyller et al. [24]. However, findings on QOL and hypertension may be biased by a underreporting. Studies by MacDonald et al. and Stein et al. showed that hypertensive patients tend to underestimate the impact of hypertension on quality of life [24,25]. This tendency of underestimation in hypertensive patients may also have occurred in our study and my have influenced the marked differences of perceived quality of life between patients with hypertension plus stroke and patients with hypertension only in our sample.

Another main finding of this study is that psychological stress was related to all domains of quality of life among hypertensive patients. This result confirms findings in a study from Ames et al. among hypertensive patients stating that quality of life is associated with psychological stress, even when accounted for age and number of chronic illnesses [15]. As stress is part of lifestyle it has been shown by McDonald that high self perceived stress was related to uncontrolled hypertension [24]. These results are important in light of potential interventions aiming at stress reduction in hypertensive CVD patients. Carlson et al. demonstrated that stress reduction enhanced quality of life in outpatients with breast cancer [26], but studies lack for the impact of such interventions on QOL in CVD patients. Thus, stress-reducing techniques might be particularly helpful in the setting for hypertensive and stroke patients aiming at a reduction of hypertension, at lowering the risk for (re-)stroke due to hypertension and subsequently to an improved quality of live [15-17].

The results from our study on the relationship between quality of life and socio-demographic characteristics among patients with hypertension plus stroke are partly contrary to findings from Wyller et al. who reported female gender and a good social network as correlates of subjective well-being one year after stroke [26]. In our study we found only particular evidence for this correlation: gender had an impact on the social domain of QOL and income on the physical domain of QOL among patients with hypertension plus stroke. Other sociodemographic characteristics such as age and education showed no significant impact on any domain of QOL among hypertension plus stroke patients.

While education had no significant impact on any of the QOL domains among patients with hypertension plus stroke, we found a statistically significant correlation between education and several domains of QOL among patients with hypertension. These results become meaningful in light of Dressler's study, who reported, that lower education was associated with high blood pressure and high mortality from cardiovascular disease [28]. According to these findings it can be hypothesized, that educational interventions on healthy life styles may play an essential part in the prevention of high blood pressure and fatal cardiovascular events.

Special characteristics of the Palestinian political and economic situation may have contributed to these findings indicating the significant relation of QOL and income and education in Gaza Strip. Unemployment and poverty are the strongest challenges hampering human development in Gaza Strip [29].

The demonstrated gab in quality of life between males and females in our study may also be due to the Palestinian male-dominant culture, but also due to additional gynecological problems, and physiological differences (menstruation, child birth, and menopause), which affect women's health both physically and mentally. The Centers for Disease Control and Prevention supports this finding with their statement that decreased health related quality of life is a particularly important issue among women [30]. In addition, Barajas et al. found that female gender and low level of education were significantly associated with worse scores of quality of life in primary care patients with obesity [31].

### Limitations of the study

The duration of treatment of hypertension or stroke was at least 1 year at the time patients were included into the study. No more medical details on the course of the disease in the previous year, on the type of brain lesion of stroke or the severity of the CVD condition potentially influencing QOL were obtained. However, for the purpose of this study which was to measure the association of psychological stress and QOL, it was important to have relatively stable medical conditions of hypertension and stroke. It was assumed that the medical condition of hypertension or hypertension plus stroke with the duration of at least one year had been chronic and relatively stable when patients were interviewed. However, we were not able to take the drugs patients were taking into account since pharmacotherapy used in hypertension or in stroke may impair quality of life.

The cross-sectional design of the study does not allow for any stable prospective conclusions on the relationship of CVD conditions and QOL, although QOL was measured after the onset of the disease.

The presented data showed that the selected variables (age, gender, income, education and psychological stress) largely explained the variance of the single domains of the QOL in hypertensive but not in hypertensive plus stroke patients. Future research could focus on further explanatory variables for quality of life in patients with hypertension plus stroke or hypertension only, such as cognitive and psychological (e.g. orientation, memory, attention, concentration, language) factors with impact on general functioning, general well-being and QOL. Previous research suggests addressing neuro-psychological features in QOL research might be beneficial as these factors are suitable for the rehabilitation of CVD patients [32].

## Conclusion

It can be concluded that QOL in patients with hypertension plus stroke is likely to be more influenced by the disabling effect of stroke itself, and it appears to be less attributable to psychological stress and sociodemographic factors than in patients with hypertension. Future research is needed to confirm these results.

As psychological stress is a modifiable risk factor, interventions aiming at stress reduction in hypertensive patients might substantially contribute to a better quality of life and to the prevention of stroke in hypertensive patients.

## Declaration of competing interests

The author(s) declare that they have no competing interests.

## Authors' contributions

BB conceived the study, designed and developed the questionnaire, performed statistical analysis and drafted the manuscript. YA conceived the study and participated in the design and development of the questionnaire, co-ordinated and carried out the data collection in Gaza and helped to draft the manuscript. All authors read and approved the final manuscript.
